# Writers' Diabetes

**Published:** 1897-01-30

**Authors:** 


### WRITERS' DIABETES.

There are, or were until quite recently, at least three editors of high-class metropolitan newspapers who are known to suffer from one form or another of diabetes, and there is reason for believing that this disease is far from uncommon, not only among editors, but also among the rank and file of journalists, whose work is not less arduous than that of editors, and whose rewards are much less substantial and consolatory.

It accords with the experience of some observers that writers as a class, but more especially journalists, are apt to suffer from glycosuria and all the train of disabling symptoms which are associated therewith. The reason for this is not far to seek. It is not work merely, nor even over-work, which is the cause. For many persons habitually work hard and long, too hard and too long, without a resulting diabetes. The work of the regular journalist is marked by these peculiarities, that it is very strenuous, that it calls for the exercise of the highest mental powers possessed by the individual, that the exercise of these powers is not intermittent and occasional, but constant, and especially that it is associated with a considerable amount of very real and deep mental anxiety. Worry of any kind, if sufficiently prolonged, is, as everybody knows, very likely to result in diabetes in persons of a certain order of constitution. The physician who sees a diabetic for the first time invariably questions the patient on the subject of past anxieties; and as, invariably, when prescribing for him, insists upon the entire avoidance of anxieties in the future. It is, then, a commonplace of the consulting-room that anxiety and glycosuria stand to each other in the relation of cause and effect. The journalist, on his part, may not often be overwhelmed with great storms of adversity and anxiety. But the probability, the almost certainty, is that his small anxieties, coupled with his very strenuous, his very thoughtful, and, above all, his very constant work, result more certainly in glycosuria than the occasional passion-storms through which many men of business are called to pass.

This is a serious outlook for the man whose sole occupation and only means of livelihood is journalism. So keen is the competition in the profession, so wide open are its portals to anybody and everybody, so uncertain is the taste of the reading public, and so doubtful therefore is the future of the average journalist, that those who depend on journalism for a living are peculiarly exposed to the nervous breakdown which follows worry, as well as to the bodily ailments which result from work.

The first aim of the writer should be to minimise the evils of his calling as far as possible, and when minimisation is not possible to counteract them. The most important minimisation is that which consists in short hours and regular meals. The most efficient counteractive is a fairly energetic open air life. If a brain-worker will begin and finish his work within as short a time as is compatible with its excellence; if he will take his meals with regularity and avoid beginning work too soon after a meal; if he will take daily two or three hours in the open air, walking, riding, cycling, gardening, or the like, spending his physical energy with strict moderation, the freshness of his health may be wonderfully preserved. If on the other hand he spends all or most of his time in close rooms, does his work by fits and starts, and takes little or no physical exercise, he courts failure of health, which must lead to failure in his profession.

The journalist's calling, from the national and medical standpoints, is not second in importance to any of the intellectual callings. There are those who hold that in national significance it takes precedence of the pulpit, and we are inclined to agree with them. Medically we know that the work of a man, but most of all his mental work, is extremely dependent upon the condition of his health. If we want a nation of men—and we do—we must, without question, have a nation of manly journalists. Here lies the importance of the subject from the national and medical points of view. Journalists cannot by any possibility be manly unless they are healthy; they cannot by any possibility be healthy unless they fulfil the ordinary conditions of health. That they do not fulfil these conditions so generally as they should is demonstrated by the wide prevalence of glycosuria amongst them.

